# Affect of Early Life Oxygen Exposure on Proper Lung Development and Response to Respiratory Viral Infections

**DOI:** 10.3389/fmed.2015.00055

**Published:** 2015-08-10

**Authors:** William Domm, Ravi S. Misra, Michael A. O’Reilly

**Affiliations:** ^1^Department of Pediatrics, School of Medicine and Dentistry, The University of Rochester, Rochester, NY, USA; ^2^Department of Environmental Medicine, School of Medicine and Dentistry, The University of Rochester, Rochester, NY, USA

**Keywords:** hyperoxia, influenza A virus, innate immunity, lung development, prematurity

## Abstract

Children born preterm often exhibit reduced lung function and increased severity of response to respiratory viruses, suggesting that premature birth has compromised proper development of the respiratory epithelium and innate immune defenses. Increasing evidence suggests that premature birth promotes aberrant lung development likely due to the neonatal oxygen transition occurring before pulmonary development has matured. Given that preterm infants are born at a point of time where their immune system is also still developing, early life oxygen exposure may also be disrupting proper development of innate immunity. Here, we review current literature in hopes of stimulating research that enhances understanding of how the oxygen environment at birth influences lung development and host defense. This knowledge may help identify those children at risk for disease and ideally culminate in the development of novel therapies that improve their health.

## Introduction

Growing evidence suggest gene–environment interactions during critical stages of development profoundly influence health later in life. This concept of “developmental origins of health and disease,” also called DOHaD, originated with a study by Dr. David Barker who showed that low birth weight correlated with increased risk of coronary heart disease in adults (1). DOHaD has now been linked to a wide variety of diseases in children and adults. Preterm birth, infection, tobacco smoke, and exposure to many inhaled pollutants can permanently impact lung development and immune function (2–4). Similarly, exposure to exogenous chemicals, malnutrition, and low birth weight correlates with poorer immune function (5–8). Even socioeconomic status and child abuse have been shown to influence a healthy lifestyle later in life (9). In 1983, the comedy movie *Trading Places* starring Dan Aykroyd and Eddie Murphy “tested” whether nature or nurture were responsible for distinguishing social hierarchy between two individuals. Although the question was never resolved in the movie, we are now beginning to appreciate 30 years later that gene–environment interactions influence children’s health, in part, through metabolic and epigenetic reprograming of cells required for organ growth, regeneration, and immunity.

The human lung is designed to efficiently exchange oxidant gases between the environment and blood, and exclude or defend against inhaled pollutants that otherwise disrupts this process. When considering gene–environment interactions that influence lung function, the transition to air at birth must surely be one of the most profound environmental changes that one will ever experience. In this singular moment, the delivery of oxygen and nutrients via the placenta is transferred, respectively, to the lung and gut. Both organs must therefore be developmentally mature and functional by this time. Proper development of the lung involves a complex set of transcription factors, morphogens, growth factors, and matrix molecules be expressed during precise developmental windows (10–13). Expression profiling studies have defined a pattern of gene expression wherein developmental genes are expressed first and genes involved in oxygen transport, protection against reactive oxygen species, and host defense are expressed near birth (14, 15). This “time-to-birth” program ensures that the lung is ready to breathe air and defend against environmental toxins at birth.

The interaction of genes with the oxygen environment at birth is disrupted when infants are born too soon. Many preterm infants develop bronchopulmonary dysplasia, a chronic form of lung disease characterized by alveolar simplification and restrictive airways (16). Mechanisms that promote BPD include genetics and maternal, fetal, or postnatal environments (17). It has been difficult to define which is most important for initiating or promoting disease, perhaps because BPD is clinically defined by the amount of oxygen used at a specific gestational age (18, 19). Fortunately, most preterm infants born >24 weeks gestation are surviving, albeit at the risk of developing a variety of lung and non-lung diseases later in life. Children born preterm often display reduced lung function, increased re-hospitalization following a respiratory viral infection, and incidence of non-atopic asthma (20, 21). They may also show neurodevelopmental delay and have greater risk for high blood pressure and heart disease as adults (22, 23). The annual cost of treating children in the United States who were born prematurely in 2005 was $26.2 billion dollars, of which 10% was just for treating infants with BPD (http://www.nhlbi.nih.gov/new/press/06-07-26.htm). Hence, there is an urgent need to understand how premature birth is a developmental antecedent of poorer health later in life.

The pathogenesis of BPD and the health sequela of survivors is a complex and poorly understood process, perhaps because it is a multi-organ disease originating from abnormal gene–environment interactions. Recognizing that there is a genetic program designed to create the lung and afford it anti-oxidant and innate immune defenses by birth, it seems rather obvious that preterm birth will disrupt the timing of when specific genetic programs need to be completed or in place to properly allow the lung to transition to an oxygen-rich environment. Therefore, identifying genetic variants that predispose to preterm birth may also identify variants that correlate with BPD. A screen of single-nucleotide polymorphisms identified two genes (CRHR1 and CYP2E1) acting in the fetus and four genes (ENPP1, IGFBP3, DHCR7, and TRAF2) in the mother that predisposes to preterm birth (24). But, interestingly none of these genes have been detected in other studies seeking to find variants that predispose preterm infants to BPD (25, 26). In fact, the few weak candidates detected in one study were not detected in another, suggesting that BPD is not entirely a genetic disorder. On the other hand, widespread methylation was detected in the blood of extremely preterm infants, suggesting that there were changes in blood cell development, composition, and perhaps immune function (27). Since these changes in methylation resolved by 18 years of age, they may not be responsible for the long-term health effects reported in people born preterm. Therefore, genetic susceptibility to BPD is more likely to represent genetic variants that modify how cells respond to an environmental stress, such as infection or the transition to air too soon.

Environmental stresses known to promote BPD include prenatal and postnatal infections, and oxygen or ventilator-induced damage to the lung. In both cases, inflammation and oxidative stress or damage to the developing lung seems to be a primary driver of BPD. Preterm infants are deficient in anti-oxidant enzymes and are therefore susceptible to oxidative stress, whether initiated by inflammation or supplemental oxygen therapies in the preterm infant (28, 29). Lungs of preterm infants are often underdeveloped and cannot adequately exchange oxygen and carbon dioxide. Supplemental oxygen supported by ventilation is often used to improve blood oxygen levels and prevent hypoxemia. However, it is now clear that high levels of oxygen can disrupt development of the lung and is a risk factor for neurodevelopmental delay, retinopathy, and probably other diseases attributed to preterm birth (30). Oxygen-induced damage can also elicit an inflammatory response, subsequently compounding the oxidative stress to the lung. Consistent with oxygen playing a role in the pathogenesis of BPD and the long-term respiratory complications associated with preterm birth, anti-oxidant therapies have proven partially effective in alleviating lung disease in humans and in animals exposed to high oxygen (31–34). Because the pathogenesis of neonatal oxygen exposure in humans and in animal models has been recently reviewed (19, 35–40), the following discusses oxygen-induced changes in lung development in relationship to how it also perturbs host response to respiratory viral infections.

## Proper Lung Development

**Figure 1:**
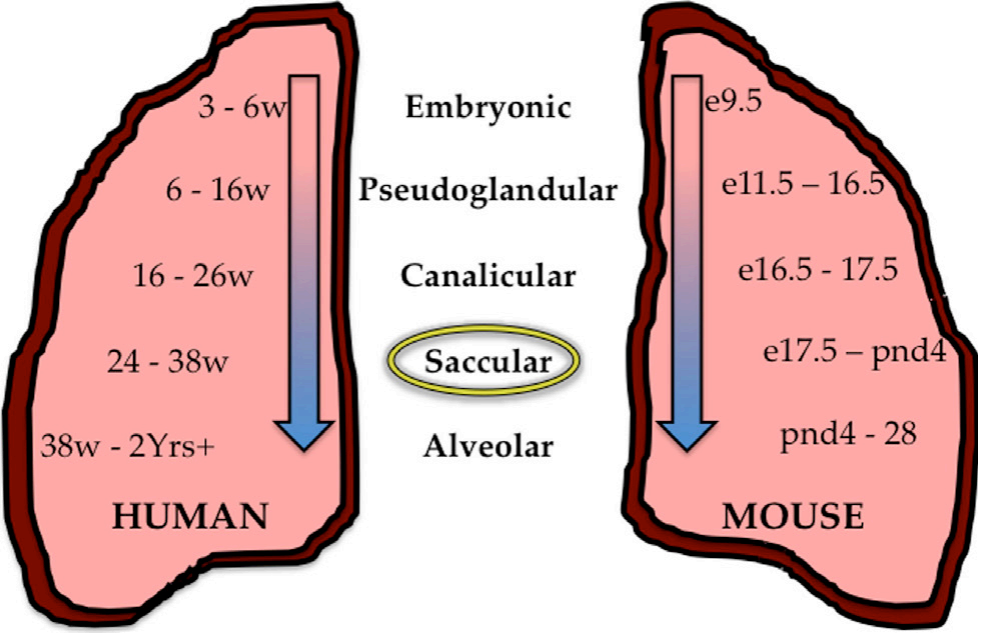
**Stages of lung development in the human and mouse**. During development, the human (mouse) lung undergoes five successive stages of development; The Embryonic stage 3–6 weeks (e9.5–11.5), the Pseudoglandular stage 6–16 weeks (e11.5–16.5), the Canalicular stage 12–26 weeks (e16.5–17.5), the Saccular stage 24–38 weeks (e17.5–PND4), and the Alveolar stage 38 weeks–2+ years (PND4–28). Preterm children who survive are often born between 24 and 38 weeks of age and are in the saccular stage of development (circled) corresponding to the saccular stage in the mouse from e17.5–PND4.

The pulmonary system, in highly simplistic form, can be described as the co-branching of air conducting and blood circulating systems that, due to simultaneous and congruent branching, efficiently interact for proper gas-exchange and subsequent systemic circulation of oxygen. In humans, gas-exchange is accomplished by diffusion through squamous epithelial cells in the alveolar saccules of the mature lung. Branching morphogenesis of the airways that concludes with formation of the alveolus leads to an impressive pulmonary surface area of around 70 square meters with a thickness of 0.1 um capable of supporting an oxygen consumption of 250–5500 ml/min (41, 42). This developmental program progresses through five successive stages. The mammalian lung undergoes five stages of maturation that begin with the embryonic stage, followed by the pseudoglandular, canalicular, saccular, and ending with the alveolar stage (Figure 1). The timing of these stages during fetal and postnatal periods varies between species, including between humans and mice. This is important when attempting to model human diseases in experimental animals. For example, many preterm infants born today are in the saccular phase of lung development, which pathologically corresponds to e17.5 to postnatal day 4 in mice. Hence, the mouse is an appropriate experimental model for studying how too much oxygen can perturb saccular development in preterm humans. Additional details on factors controlling lung development have been reviewed elsewhere (10, 12, 41, 43).

Successive developmental stages are defined by changes in lung morphology. In the embryonic stage, the pulmonary branching pattern originates and two distinct lobes are formed. The pseudoglandular stage marks the appearance of numerous terminal buds projecting away from the initial two lung lobes and recent work has defined the patterns as domain branching, planar, and orthogonal bifurcation budding (44). During the canalicular stage, epithelial tubules form with large terminal buds while the mesenchyme separates into dense subsets between future alveolar septa. Specialized epithelial cell types and alveolar sacs emerge during the saccular stage of development. Squamous type I epithelial cells form the lining of the alveolar sacs with cuboidal type II epithelial cells interspersed. Thinning of the mesenchyme along with an increase in extracellular matrix allows for expansion of these alveolar sacs culminating in the alveolar stage where dense connective tissue, containing cartilage and smooth muscle, surrounds the airways. The timing of developmental completion, leading to the formation of alveolar sacs, varies between species. In mice and rats, alveolar development concludes mainly postnatally characterized by lung expansion and alveoli subdividing into smaller gas-exchanging units (45). Importantly, this morphogenic process has been accompanied by blood vessel morphogenesis that concludes with capillary networks residing in close proximity to the alveolar epithelium.

It is often written that the normal adult mammalian lung contains approximately 40 different cell types, yet the origin of this statement seems to have disappeared in the historical literature. However, it should not be surprising to find that this is a gross underestimation when one considers how expression of cell surface receptors has markedly increased the diversity of leukocytes present in the lung (46). The emerging use of microfluidic single-cell RNA sequencing is also uncovering an equally rich diversity among non-hematopoietic cell populations (47, 48). Pulse-chase labeling with H-thymidine, cell-restricted fluorescent reporter genes, and cell-specific ablation with toxins has identified region-specific niches containing stem cells required for proper lung development and repair (49). Unique specific stem cell niches may therefore have evolved to facilitate repair of specific areas of the lung damaged by region-specific toxins. Since perinatal exposures influence saccular and alveolar phases of development, the following briefly focuses on progenitor cells controlling distal airway and alveolar development and regeneration.

The region where the airway meets the alveolus has been termed the bronchoalveolar duct junction (BADJ) (50). The distal airway epithelium contains Clara (now called Club) cells defined by their cuboidal appearance and expression of secretoglobin family 1A, member 1 (Scgb1a1), also called Clara Cell Secretory Protein (CCSP) or uteroglobin. During recovery from naphthalene depletion, a population of Club cells proliferates from neuroendocrine bodies and from the BADJ (51, 52). These bronchoalveolar stem cells (BASC) express airway Scgb1a1, alveolar Type II surfactant protein (SP)-C, the stem cell markers Sca-1, and CD34, but not CD45 (53). These BASCs are able to self-renew and maintain expression of both airway Scgb1a1and alveolar SP-C expression when cultured on irradiated mouse embryonic fibroblasts. However, their importance in defining airway and alveolar epithelial cell development and repair remains unclear because they proliferate less frequently than Type II cells in a post-pneumonectomy model of lung regeneration (54).

A label-retaining population of airway cells expressing Scgb1a1 and the stem cell markers Oct-4, Sca-1, and SSEA-1 has also been identified in BADJ (55). These cells can be maintained *ex vivo* for several weeks, but have the capacity to express SP-C and T1α when cultured on Type I collagen. Fate-mapping studies using Scgb1a1-driven reverse transcriptional transactivator (rtTA) gene or Cre fused to an estrogen responsive binding site (CreER) gene to durably label Scgb1a1+ cells with LacZ or fluorescent proteins has provided new insight into the ability of airway Scgb1a1+ progenitors to repopulate alveolar cells. Depending upon the model and the timing of activation, airway Scgb1a1+ progenitors contribute to ~10–50% of adult type II cells during normal postnatal lung development (56–60). These cells also contribute to alveolar repair when adult mice are infected with Influenza A Virus (IAV) or injured with bleomycin, both of which damage alveolar type II cells (59). Interestingly, they do not participate in repair when mice are exposed to hyperoxia or naphthalene (58). Since hyperoxia injures alveolar type I cells, and naphthalene injures airway Club cells, these two studies suggest Scgb1a1 + cells may serve as precursors for themselves and type II cells.

Analogous to studies using naphthalene to ablate airway Clara cells, exposure of adult mice, rats, or monkeys to oxidant gases (hyperoxia, ozone, or nitrogen dioxide) kills alveolar type I epithelial cells (61–63). Pulse-chase labeling studies with H-thymidine indicate type II epithelial cells proliferate and differentiate into type I cells following injury (64–66). Emerging evidence suggests that subpopulations of type II cells exist and T1α, a protein expressed by Type I cells, has been shown to co-localize with the Type II cell-specific lectin Maclura pomifera (67). Tri-transgenic mice containing the rat airway CCSP promoter driving rtTA, the otet-Cre gene, and the LacZ/EGFP (Z/EG) reporter identified a lineage of epithelial cells that defines airway Club and a small population of alveolar Type II cells (68). Recently, single-cell RNA sequencing revealed the existence of four distinct populations of type II cells (48). Alveolar type I cells have historically be thought to be the most terminally differentiated cell of the lung whose sole function was to facilitate gas-exchange and maintain barrier function (64–66). However, a study showing that type I cells isolated from rats can proliferate *ex vivo*, express the stem cell protein Oct-4, and can be induced to express SP-C and Scgb1a1 has challenged this conclusion (69).

## Pulmonary Response to Influenza a Infection

As the lung evolved to efficiently exchange oxygen and carbon dioxide, so did an innate immune system comprised of specialized epithelial resident cells and circulating immune cells that function to recognize and clear a variety of inhaled pathogens and toxicants. Failure to detoxify the airspace can result in significant disease and even death. These defenses are most likely designed to respond to inhaled pathogens, like respiratory viruses, which were present in the environment before vertebrates migrated onto land. We therefore will discuss the current understanding of the pulmonary interactions with respiratory infections, primarily focusing on IAV, in an attempt to build a greater understanding of the poor response experienced by children born prematurely.

Viral respiratory infections have been found to afflict preterm infants at a higher rate than full term controls. Respiratory Syncytial Virus (RSV), human Rhinovirus (RV), and Bocavirus infection of children less than 14 years of age hospitalized over a 7-year study period were described (70). The authors found that children who were preterm exhibited a higher rate of infection with human metapneumovirus and parainfluenza virus as compared to controls (70). Additionally, a recent study describes extremely and moderately preterm infants facing a 3.6 times increased risk of being hospitalized due to respiratory infection, likely from RSV or RV, in the first year of life (71). Preterm infants hospitalized due to RSV were found more likely to wheeze in the first six years of life and experience decreased quality of life versus those infants who were not hospitalized due to RSV infection (72). RV infection of preterm infants also increases the risk of developing wheeze and requiring respiratory medicines in the first year of life, and can be the source of serious lower respiratory tract infections (73–76). A recent NHLBI workshop report recommends identifying prophylactic approaches to prevent RSV and RV infections to help lessen the burden of asthma development in childhood (77), however determining when the use of such prophylaxis is complicated (78). Thus, infants born preterm face serious consequences in response to respiratory viral infections.

In human pediatric populations, RSV is more common in infancy (first two years of life) while IAV is generally more common in school age children (79, 80). Gaining a better understanding of how early life oxygen exposure affects responses to respiratory viral infections necessitates the use of animal models. While different species have shown utilization in RSV modeling, each has advantages and disadvantages (81, 82). Human RSV does not efficiently replicate and leads to non-significant disease and mortality in mouse models, making it difficult to model how it is perturbed in preterm children (82). This is in contrast to IAV mouse models that have proven robust viral replication and disease that closely model human disease. Here, neonatal oxygen exposures that have been shown to promote BPD-like lung disease in mice have also been shown to alter the response to IAV infection (35). Understanding how the oxygen environment at birth disrupts the host response to IAV may provide insight into how it influences the response to RSV and other respiratory viruses.

IAV annually causes global seasonal epidemics but also novel IAV occasionally arise leading to global pandemics. The most notorious of which was the pandemic of 1918 and the most recent the 2009 swine-flu pandemic (83). Significant insight into IAV–host interactions has historically occurred through *in vitro* investigations. A much greater understanding of this virus–host interaction, prior to, during, and following significant pathological outcomes *in vivo*, has been hampered due to a lack of traceable reporter expressing IAV that retain full virulence as well as other technical problems. Recently, IAV–host interactions and *in vivo* dynamics following infection have been investigated utilizing reporter expressing recombinant IAV (84, 85).

The first step in IAV infection involves the recognition of sialic-acid (SA) moieties on the surface of susceptible cells by the viral hemagglutinin (HA) protein. Human IAV primarily infect via α2-6 SA residues and avian IAV by α2-3 linked residues. In healthy humans, α2-6 SA has been primarily found on the epithelial (ciliated and non-ciliated) and goblet cells of the upper respiratory tract in humans (86). Avian like α2-3 SA has primarily been found on non-ciliated bronchiolar and alveolar type II cells in the lower respiratory tract (86, 87). Viral attachment and histochemical studies have revealed human IAV primarily interacting with the upper respiratory tract through ciliated epithelial cells, goblet cells, as well as to type I alveolar epithelial cells, to varying extents (86–89). Contrasting with human IAV, avian IAV has been shown to primarily attach to alveolar epithelial type II cells, alveolar macrophages, and bronchiolar non-ciliated epithelial cells (89, 90). Sialic-acid receptor expression is a good correlate of IAV binding based upon histochemical studies. Although human IAV is of primary concern for understanding infection of the population discussed in this review, understanding avian IAV infection is imperative in the face of novel viruses entering the human population.

Both human and avian IAV can infect human airway epithelial cultures with human IAV preferentially target non-ciliated airway cells whereas avian IAV infect ciliated populations (91, 92). Alveolar type II cells have also been demonstrated as a site of IAV infection and replication although their importance to human disease is currently unclear. Human alveolar type II cells were infected by IAV in a primary cell culture system (93). Alveolar type II cells are imperative for the maintenance of the alveoli by producing and secreting surfactant as well as being a renewable source for themselves and type I alveolar cells. Although poorly understood, the affect of IAV infection of type II cells has been shown to affect their phenotype and subsequent innate immune responses (93). Taken together, IAV tropism as it relates to human disease requires further investigation. Differences based on the strain of IAV used and type of assay utilized must be clarified for a greater understanding of human disease.

The source of cells responsible for pulmonary regeneration following viral injury is currently an active area of research. Bronchiolar epithelial cells expressing p63 were found to rapidly expand and disseminate to areas of lung injury following IAV infection and repair (94). This cell population was also found to have the ability to form “pods” in both bronchiolar as well as alveolar regions following injury caused by IAV. Keratin 5 expression (Krt5) was also shown to map to these regions and, importantly, was only detected following IAV infection, during reparative processes (94). These p63/Krt5 + cell populations therefore may act as distal airway stem cells and serve as the source for alveoli cell regeneration following injury and recently these p63/Krt5 + cells were found to recapitulate alveoli following epithelial injury by IAV (95). This unique population also has the ability to form alveoli-like structures when delivered to IAV-infected lungs minimizing virus-induced pathology (95).

## Oxygen Perturbation of Proper Lung Development and Innate Immunity

**Figure 2:**
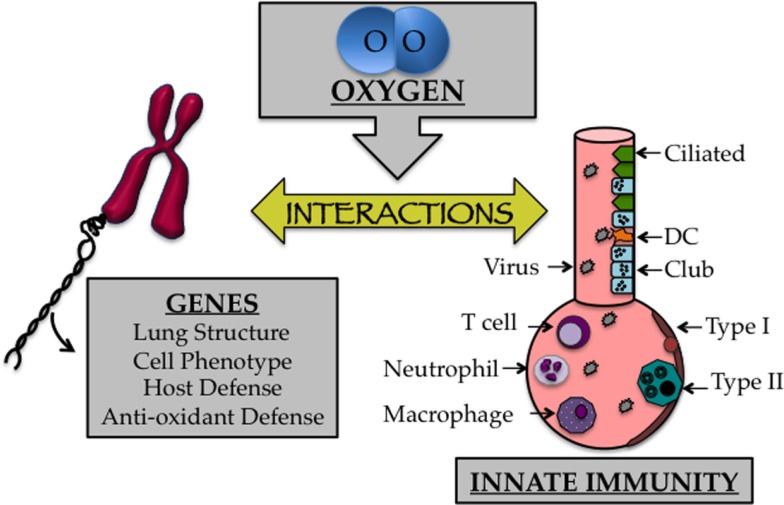
**The early life oxygen environment affects changes in genetic as well as innate immune mechanisms**. Cartoon depicting the affect early life oxygen environment imparts on genes that specify lung structure and function with cells involved in innate immunity.

As discussed previously, the transition to an oxygen environment at birth may be one of the most profound environmental changes one will ever experience and can lead to disease when it occurs inappropriately. Lungs of infants born preterm are often in the saccular phase of development. Alveolar regions at this time have yet to develop into true gas-exchanging structures, which is why many preterm infants develop respiratory distress. Furthermore, the capillary network surrounding the alveolus, which shuttles oxygen to the circulation, has yet to effectively complement the alveolus (96). Despite the life-saving efficacy of supplemental oxygen treatment during this critical time, growing evidence suggests that this treatment contributes to bronchopulmonary dysplasia (BPD), a chronic lung disease that is characterized by alveolar simplification and restrictive airways (16, 30). Oxygen-dependent changes in genes specifying lung structure and cell phenotype are likely to impact cells and molecules involved in innate immunity required for a proper host response to respiratory viral infection (Figure 2). This includes alveolar epithelial type II cells, goblet cells, eosinophils, macrophages, dendritic cells, T cells, B cells, and innate lymphoid cells, in addition to soluble mediators produced by these cells, including SPs, cytokines, chemokines, and mucus proteins mediate innate immunity (97–100). In other words, early life oxygen exposure or other oxidative stresses may drive the development of long-term lung disease by disrupting a delicate balance of cell communication between genes controlling lung development and innate immunity.

**Figure 3:**
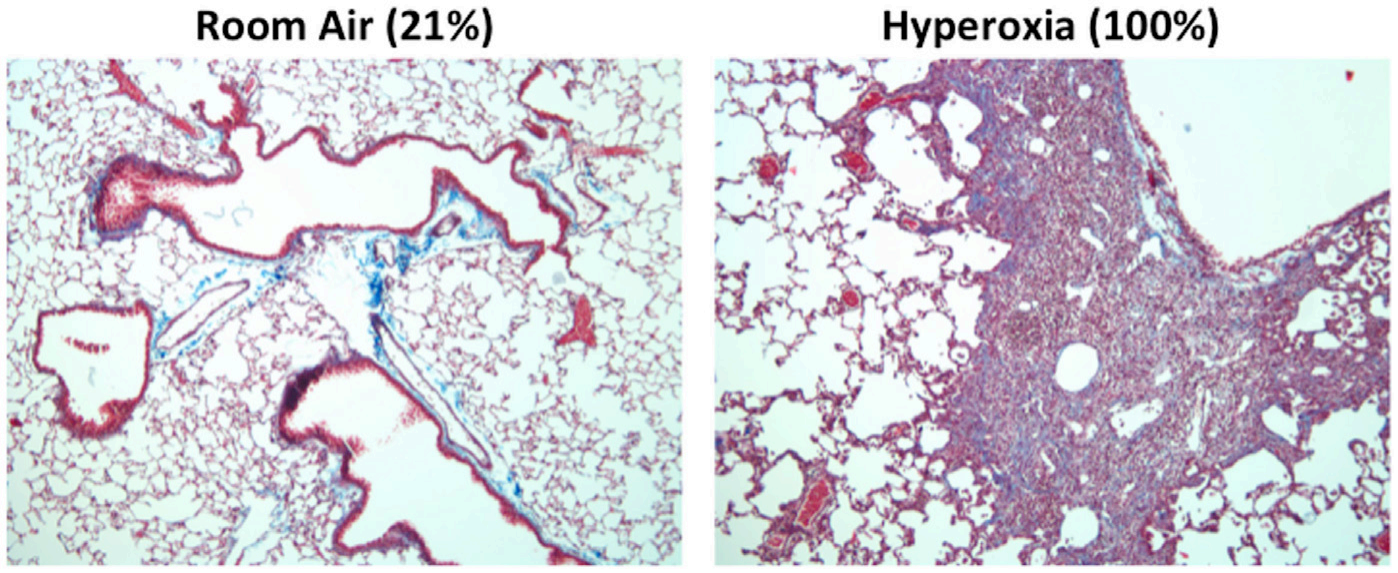
**Mice exposed to hyperoxia at birth develop fibrosis after influenza A infection**. Adult (8-week old) C57Bl/6J mice exposed to room air (21% oxygen) or hyperoxia (100% oxygen) between postnatal days 0–4 were infected with 120 HAU of influenza A virus (H3N2). Trichrome staining revealed extensive collagen deposition and inflammation in infected mice exposed to neonatal hyperoxia 14 days post infection. This pathology was not evidence in infected siblings exposed to room air at birth.

One hallmark of supplemental oxygen treatment at birth is the development of a highly simplified alveolar epithelium. Although incompletely understood, this may develop due to oxidative stress or an aberrant immune response that suppresses angiogenic factors (101). In mice, alveolar epithelial type II cells expand rapidly following neonatal hyperoxia compared to room air control littermates (102, 103). Following recovery in room air however, this population is significantly pruned (102, 103). This results in a significant decrease in the pool of alveolar type II cells later in life. Concomitant with the loss of type II cells, markers for type I alveolar epithelial cells increase during the same time frame. Currently, the source of these cells is unclear; however, evidence suggests that type II alveolar cells lost during recovery in room air are not the source of these cells (102). Further fate-mapping studies of type II and type I cells during and following exposure to hyperoxia should help to clarify the intricate balance and source of these cells. Regardless, the loss of type II cells may adversely impact alveolar repair as well as the production of innate immunity. Indeed, adult mice exposed to hyperoxia exhibit persistent and altered immune responses, fibrosis (Figure 3), and increased mortality compared to room air littermates when infected with a sublethal dose of IAV (32, 104, 105). The altered host response was not attributable to CD8 T cells and therefore the pathology is not likely due to a defect in viral clearance (106). While reduced numbers of type II cells did not negatively impact surfactant pools (107), it reduced expression of the antiviral protein eosinophil-associated RNase 1 (Ear1) detected in some type II cells (104). Reduced expression of Ear1, while conceptually attractive, does not solely account for the fibrotic phenotype observed in IAV-infected mice that have been previously exposed to hyperoxia as neonates. This is because neonatal hyperoxia has also been shown to enhance the severity of fibrosis in the neonatal hyperoxia model following bleomycin administration (108). Hypothetically, the loss of some type II cells may impact the orderly innate immune response releasing cytokines, chemokines, and SPs that are the first responders following IAV infection (109).

One example is monocyte chemoattractant protein-1 (MCP-1), which has been found to be selectively increased following IAV infection in a model of neonatal hyperoxia (105, 110). MCP-1 plays important roles in the recruitment of monocytes, T cells, and NK cells to sites of infection and has been shown to protect against viral and bacterial challenges (111, 112). However, aberrant MCP-1 control has also been associated with lung disease in children and adults (113, 114). While MCP-1 is an attractive target, it has recently been shown that MCP-1 is not solely responsible for the enhanced respiratory sequelae observed following IAV infection in neonatal hyperoxia-treated mice (105). This suggests that increased MCP-1 production may be an effect rather than a driver of the mechanisms leading to enhanced respiratory disease due to neonatal oxygen exposure.

In addition to an imbalance in alveolar type II cells, there are many other pulmonary innate immune mechanisms that might be affected by oxygen at birth. Animal models have identified several innate immune factors common in BPD-like lung injury. These include alterations in IL-6, IL-8, TNF-α, TGF-β, macrophage inflammatory factor-1α, IL-1β, MCP-1 MCP-2, CXCL-1, and CXCL-2 (115). Recent work also has identified mast cells as being present in the lungs of pediatric subjects who were diagnosed with BPD prior to death (100). Members of the IL-6 cytokine family have been shown to have fibrotic potential, which could contribute to lung disease (116). The compliment subunit C5a plays a role in neutrophil recruitment to the mouse lung following IAV infection and may be a potent inducer of hyperoxia-mediated lung injury via recruitment of macrophages, neutrophils, and lymphocytes, and increased expression of IL-6, TNF-α, and MCP-1 occurs (117, 118). Furthermore, C5a has been shown to increase TGF-β1 in primary human small airway epithelial cells, which could then contribute to the development of fibrosis (119). Thus, multiple factors could lead to the accumulation of C5a, which could induce inflammation in the lungs of preterm infants. Some of these factors have been proposed targets to prevent the development or to treat patients with BPD (120).

Several recent studies illustrated effects that hyperoxic stress imparts on the innate immune system. For instance, macrophages exposed to hyperoxic conditions experience cell cycle arrest and showed impaired phagocytic and chemotactic activity (121, 122). GM-CSF is critical for the maintenance of alveolar macrophages and hyperoxic stress has been demonstrated to decrease levels of GM-CSF via destabilization of mRNA in primary AEC cell cultures (123). Other studies indicate that the decrease in GM-CSF mRNA is due to upregulation of the microRNA molecule, miRNA 33 (124). This same publication illustrates the complex nature of hyperoxia by demonstrating that T cells actually up-regulate GM-CSF in response to hyperoxic stress (124). Taken together, this highlights the critical importance of macrophage balance on phenotype.

Macrophages have been shown to play a role in the development of alveoli (125). If these cells become more inflammatory in nature, such as experienced due to hyperoxic stress, they likely will contribute to lung pathology (126). A recent study illustrates that overexpression of TGF-β1 in the lung leads to the accumulation of inflammatory macrophages in a TGFβR2-dependent manner (127). Given the role for alveolar macrophages in activating T cells, it is possible that regulatory function of CD4 T cells could be compromised by pro-inflammatory macrophages found in the lung (128). In fact, active research is being conducted to try and target inflammatory macrophages to treat lung disease (129).

Neutrophils play a prominent role in the pathology of many lung diseases, including BPD (130). In a mouse model of hyperoxia, histological damage is preceded by neutrophil infiltration into the lung following a wave of macrophage recruitment (131). Several studies using animal models of hyperoxia show that reducing neutrophil infiltration correlates with decreased lung disease (132–134). Neutrophils play a complex role in the mechanism of inflammatory disease and it has recently been suggested that neutrophils can play an anti-inflammatory role in addition to their common pro-inflammatory role (135).

Human studies have reported an unexpected alteration in neutrophil counts in preterm infants, which could relate to the risk of preterm infants developing lung disease (136–138). Of note, one study reports that infants with respiratory distress syndrome born less than 32 weeks gestational age who develop BPD have elevated levels of IL-6 and IL-8 in tracheal aspirates prior to the influx of neutrophils versus those who do not develop BPD (139). A decrease in CD18 and CD62L on circulating neutrophils in the first 4 weeks of life in preterm infants was associated with the development of BPD (140). Additionally, increased serum levels of neutrophil-associated gelatinase-associated lipocalin in preterm infants born less than 31 weeks of gestation was predictive for the development of BPD (141). Of note, children who were born less than 32 weeks gestational age have higher IL-8 and neutrophil cell counts in sputum at the preschool age, which illustrates long-term consequences in lung inflammation due to preterm birth (142). Thus, more studies are needed to understand how hyperoxia could alter the function of neonatal neutrophil function, which could then affect the development of inflammatory lung disease later in life.

It is becoming more apparent that respiratory disease pathology varies greatly and that unique subtypes of disease exist. Many of these subtypes display unique alterations in the skewing of the immune system toward a Th1, Th2, or Th17 response (143). The endotype of disease tends to track with the type of T cell skewing with a Th17/neutrophilic response being more damaging than other types of disease, and this is intimately related to the stimulatory conditions of activated T cells (144). In a study of extremely preterm infants (born <32 weeks GA) RV infection was shown to induce a Th2 and Th17 response, and IL-4 production was related to severity respiratory morbidity (145). Furthermore, alterations in T regulatory cells have been described in humans with respiratory disease (146). An important consideration is that T regulatory cells are associated with inhibition of fibroblast proliferation and in vascular repair in the lung following injury (144). Given the surprising finding that cord blood contains T cells with an activated/memory phenotype, it is possible that these cells are poised to contribute to inflammatory lung disease (147). Recent work has also reported decreased CD4 T cells in cord blood from preterm infants who develop moderate BPD (148). Despite the finding that cytotoxic T cell function is not altered in mice exposed to hyperoxia followed by IAV infection (106), it is possible that CD4 T cells play a role in hyperoxia-mediated lung damage in humans and in the development of disease later in life. However, small animal models of oxygen effects on BPD do not support this hypothesis.

In adults, oxidative stress plays a role in COPD disease progression (149). It is possible that changes in the oxidative state of the lung due to chronic oxygen exposure in preterm infants could change how cells from the immune system respond to environmental exposures by altering cellular function or the types of cytokines that are produced (150–153). These cytokines could work in concert with cell types in the lung, including epithelial cells and innate lymphoid cells, known to produce pro-inflammatory and pro-fibrotic factors under certain conditions (154). One recent report demonstrates that reactive oxygen species in the lung can alter signaling of the inflammasome, leading to increased inflammation (155). One cell lineage receiving a great deal of attention is the innate lymphoid cell, which is a bone-marrow derived population found at mucosal surfaces, including the lung. They have the ability to generate high levels of cytokines that can influence the balance of the immune system (156). Much like cells in the adaptive immune system, they can be skewed to express transcription factors and produce cytokines consistent with Th1, Th2, and Th17 CD4 T cell lineages and play an essential role in responding to infection (157, 158). Of particular interest, ILC2 cells have been shown to play a role in the pathogenesis of lung disease by contributing to a Th2 T cell response (159, 160). IL-13 is a Th2 cytokine that, when overexpressed in the lung, results in oxidative damage to peripheral blood cells (161). Of note is that oxidized guanidine perpetuates the inflammatory response (162). A related inflammatory mechanism could be present with complexes of oxidized high-mobility group box protein 1, which has been shown to induce hyperoxia-mediated lung inflammation (136, 163). It is tempting to speculate that exposure to hyperoxia could contribute to this inflammatory loop of chronic lung disease through the induction of oxidized DNA.

Taken together, the balance of redox state within the lung is of critical importance in preventing chronic lung disease. It is very likely that early life exposure to hyperoxia changes this balance, which could result in permanent lung injury. Alterations in function of immune cells, including but not limited to CD4 T cells, neutrophils, and macrophages, likely play a major role in this development of lung disease. Importantly, pulmonary cells that produce innate immune molecules, like type II epithelial cells, might also be depleted or epigenetically modified in their ability to respond to injury (102). Taken together, it is likely that low levels of inflammation are present following exposure to hyperoxia, which could perpetually contribute to lung disease.

## A Perspective on Oxygen as a Goldilock’s Modifier of Respiratory Health

If we accept that high levels of oxygen at birth can alter children’s health, does low levels of oxygen at birth also affect children’s health? Indeed, there is growing evidence that gene–environment interactions influences health of people living at high altitude (low oxygen). Populations of Tibetans, Ethiopians, and Andeans living at >2.5 miles or between 11 and 13% oxygen exhibit resistance to hypoxemia, and develop larger lungs and hearts. These phenotypic changes appear to be genetically fixed in Tibetans and Ethiopians, but not in Andeans. Between 2010 and 2014, single-cell gene analysis and whole-exome sequencing identified haplotypes in the prolyl hydroxylase EGLN1, hypoxia-inducible factor (HIF)-2α, and peroxisome proliferator-activated receptor (PPAR)-α genes that correlated with lower hemoglobin levels in Tibetans (164–167). These haplotypes are not detected in Ethiopians. Instead, haplotype changes in the retinoic acid orphan receptor have been detected, which is interesting because this receptor dimerizes with HIF-2α (168). Taken together, this suggests that Tibetan and Ethiopian populations adapted separately to hypoxia through a common EGLN-HIF signaling pathway. Genetic changes conferring resistance to hypoxia have yet to be detected in Andeans and the hypoxic-resistant phenotype is only present in children born at high altitude (169). This implies Andeans acclimatize to an environmentally low level of oxygen at birth.

Regardless of how adaptation at high altitude is achieved, it maladaptively influences long-term health. When compared to people living at sea level, high-altitude natives have increased risk for cardiovascular disease particularly related to cardiac hypertrophy (169). A zip code study of children born at high altitude in Colorado suggests that birth at high altitude increases re-hospitalization following infection with RSV (170). Living at high altitude may also reduce brain activity (171). High-altitude natives may have lower rates of obesity (172), but are often born small for gestational age and exhibit transient growth delay with compensatory catch-up growth (169, 173). Some of these health risks may mirror those seen in children who had sleep apnea, placental insufficiency, or cyanotic congenital heart disease as infants. Hence, adapting to low oxygen at birth causes similar maladaptive changes to children’s health as high oxygen exposure.

**Figure 4:**
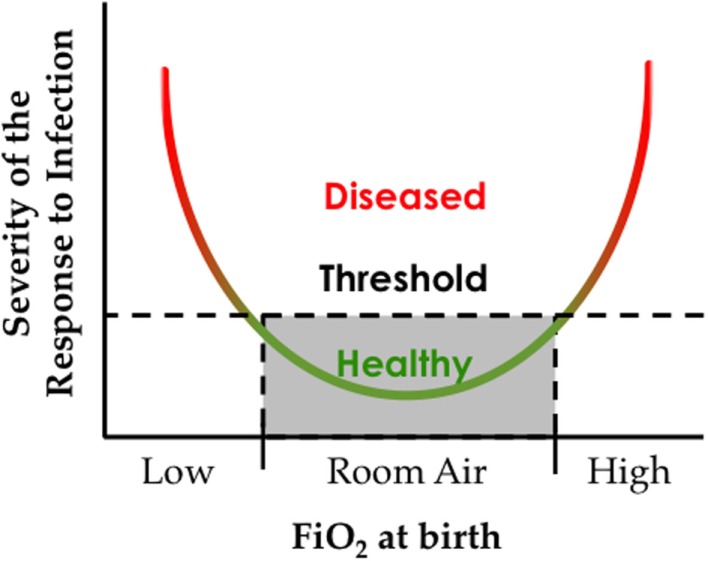
**The oxygen environment at birth affects the severity of respiratory viral infection later in life**. Hypothetical graph depicting how exposure to low or high inspired oxygen at birth can increase respiratory morbidity following a respiratory viral infection.

This Goldilocks effect of oxygen reflects the convergence of an oxygen environment on genes present at birth, some of which have fixated changes that maintain the response to hypoxia even at sea level. Genetic changes that influence the response to high oxygen used to treat preterm infants have yet to be identified, perhaps because there is no evolutionary pressure or memory for adapting to hyperoxia. However, recognizing that the response to oxygen is non-linear, studying adaptation to low oxygen may help us understand adaptation to high oxygen (Figure 4).

In the preceding sections, we have highlighted the current understanding of normal pulmonary development and how it is perturbed due to premature birth. These changes become exasperated due to neonatal oxygen exposure that affects the pulmonary epithelium, angiogenesis, and the innate immune system in the developing infant. Great strides have recently been realized in both the treatment and understanding the mechanisms leading to sequelae later in life in this susceptible population. Our hope is that this review has left the reader with an appreciation for previous work as well as highlighting future areas of research that are warranted. These include but are not limited to gaining a more complete understanding of the molecular programing that drives development and regeneration of the respiratory epithelium that will allow for a better appreciation of the affects an immature lung experiences due to premature birth into an oxygen rich environment. Infants born prematurely, and likely provided oxygen, experience enhanced disease due to respiratory infections later in life. Understanding what pulmonary cell types are principally infected by various respiratory pathogens, like IAV, in healthy subjects precludes our understanding of the cell-specific alterations occurring in preterm infants later in life. Although cell-specific pulmonary tropism of IAV is unlikely to drastically change in this population, it may prove that a cell-specific imbalance in these aberrant lungs drives enhanced disease. It is also clear that genes involved in directing lung development overlap with those of the pulmonary innate immune system (97). It is therefore likely that overall respiratory health is accomplished by an interaction with oxygen at birth that influences the developmental trajectory of the lung and pulmonary innate immune system. A better understanding of how the oxygen environment at birth influences gene–innate immune interactions could help identify children at risk for disease and ideally treatments that improve their health.

## Author Contributions

The design, writing, and editing of this manuscript was done with equal participation and intellectual contributions by WD, RM, and MO. Final editing and manuscript preparation was performed by WD.

## Conflict of Interest Statement

The authors declare that there are no commercial or financial relationships serving as a conflict of interest in the writing of this manuscript.
